# The association between remnant cholesterol and rheumatoid arthritis: insights from a large population study

**DOI:** 10.1186/s12944-024-02033-z

**Published:** 2024-02-07

**Authors:** Yuxin Yan, Rui La, Ming Jiang, Wu Xu, Dinghua Jiang, Shenghao Wang, Lixin Huang, Qian Wu

**Affiliations:** 1 Department of Orthopedic Surgery, Institute of Orthopedics, The First Affiliated Hospital of Soochow University, Soochow University, Jiangsu, China; 2 Department of Orthopedic Surgery and Biochemistry, Jeonbuk National University Medical School, Jeonju, Republic of Korea

**Keywords:** Remnant cholesterol, Rheumatoid arthritis, National health and nutrition examination survey, Cross-sectional study

## Abstract

**Objectives:**

While lipid metabolism disorder is widely acknowledged as a contributing factor to inflammation, the association between remnant cholesterol (RC), which indicates lipid metabolism, and rheumatoid arthritis (RA) has not been investigated. Accordingly, this study evaluated whether RC is associated with RA disease events.

**Methods:**

Data were collected and specifically extracted from the National Health and Nutrition Examination Survey (NHANES) 1999–2008 database. The RC value was derived by subtracting the combined amount of low-density lipoprotein cholesterol (LDL-C) and high-density lipoprotein cholesterol (HDL-C) from the total cholesterol (TC). The association between RC and RA was evaluated using multivariate regression analysis and subgroup analysis.

**Results:**

The study analyzed 7777 patients, of which 581 patients (7.47%) were diagnosed with RA. After accounting for different covariates, the multivariate logistic regression analysis revealed a notable correlation between increased RC levels and an increased likelihood of RA (odds ratio OR = 1.54; 95% confidence interval CI: 1.11–2.13; *P* = 0.0092). The interaction test did not yield statistically significant effects on this association. The linear correlation between RC and RA was observed within restricted cubic spline regression model limitations.

**Conclusion:**

The results suggest that higher RC levels are associated with increased odds of RA, indicating that RC can serve as a novel and convenient index for forecasting the likelihood of RA in the United States. Additionally, these findings offer insights into early intervention strategies for susceptible populations at risk of developing RA.

## Introduction

Rheumatoid arthritis (RA) is a long-lasting autoimmune disorder that primarily impacts the synovial joints, leading to persistent pain, inflammation, and stiffness. This condition significantly affects a person’s overall health, ability to move, and quality of life [1]. The Global Burden of Disease study highlighted that nearly 18 million people, or approximately 0.6% of adults globally, have RA [2]. Without medical intervention, up to 30% of RA patients face permanent work disability within 2–3 years of diagnosis [3]. Although RA progression is involved in lipid metabolism in autoimmune diseases, its etiology has not been fully elucidated. The precise cause of RA remains uncertain but likely stems from modifiable and non-modifiable factors combined, including diet, environmental exposures, genetics, sex, and age [4]. Individuals diagnosed with RA frequently display atypical lipoprotein profiles, marked by diminished levels of high-density lipoprotein cholesterol (HDL-C) and heightened levels of apolipoproteins A-1, B, and E [5]. RA is associated with an abnormal lipoprotein pattern, principally low HDL-C [6]. RA is also associated with elevated levels of lipid derivatives such as leukotriene B4 originating from arachidonic acid and contributing to the pro-inflammatory environment observed in RA [7]. Additionally, RA is characterized by lipid derivative imbalances, with increased pro-inflammatory leukotriene B4 and reduced pro-resolving lipid mediators such as resolvins D3, D4, and E3 [8]. Despite these valuable insights, these lipid metabolism markers are not commonly tested in the clinic.

Remnant cholesterol (RC) is an emerging tool representing cholesterol in triglyceride-rich lipoproteins and is implicated in a spectrum of conditions, encompassing cardiovascular and metabolic disorders [9]. Serum lipids have emerged as potential biomarkers for various diseases, including cardiovascular and cerebrovascular diseases [10, 11], metabolic diseases [12], infectious diseases [13], hematological diseases [14], and cancer [15]. Most previous studies on cholesterol metabolism focused on low-density lipoprotein cholesterol (LDL-C) and HDL-C. RC was recently demonstrated to equally precisely predict atherosclerotic cardiovascular disease (CVD) risk compared to LDL-C or very low-density lipoprotein (VLDL) [16]. The variability in RC values has been consistently correlated with an elevated probability of experiencing ischemic stroke within the broader population [17, 18], and beyond the impact of LDL-C, RC was found to be linked with hypertension in the adult population in the United States as a whole. This association persisted even when considering elevated triglycerides (TG) levels, suggesting the involvement of lipoproteins other than apoprotein B [19]. A few preliminary studies have suggested that RC may serve as a potential risk factor for hypertension and type 2 diabetes mellitus [20]. Consequently, RC has emerged as a critical parameter in lipidology and is subject to ongoing research to uncover its broader clinical applications. However, no study has reported whether RC is associated with RA or how.

Furthermore, there is a lack of studies utilizing the National Health and Nutrition Examination Survey (NHANES) database to investigate the relationship between RC and RA. The objective of this study was to elucidate the correlation between RC levels and RA among NHANES participants. It was hypothesized that individuals with RA would exhibit elevated RC levels.

## Methods

### NHANES study population

The NHANES database is an all-encompassing study conducted in the United States that collects vast information on the overall health and nutritional well-being of the general populace. The NHANES uses meticulously structured stratified multistage random sampling to ensure accurate representation. Furthermore, the NHANES is ethically sanctioned by the National Center for Health Statistics, adhering to rigorous ethical standards. The survey mandates informed consent from all participants, underscoring its commitment to upholding ethical principles in research involving human subjects. Access to NHANES datasets, including comprehensive documentation and protocols, is available for free via the website.

This study used the NHANES 1999–2008 as it encompasses data on RC and RA, which are the focal points of the present study. The participant exclusion criteria were used to guarantee the validity and reliability of the results and were as follows: (1) participants with missing data on total cholesterol (TC), LDL-C, and HDL-C were filtered out as these variables were integral to the calculation of the RC; (2) participants with incomplete data related to arthritis were excluded from the study to mitigate potential biases and enhance the robustness of the analysis; (3) participants with osteoarthritis (OA) and those with other non-applicable conditions were deliberately excluded from the study, ensuring that the analysis specifically focused on participants meeting the parameters of interest; (4) pregnant women, whose metabolic profiles can change during pregnancy and might affect the analysis, were also excluded.

### Assessment of RC and RA in NHANES

The calculation of the RC is a process that entails determining its specific value by subtracting both the HDL-C and LDL-C values from the TC value. Consistent with the previous investigation [21–23], arthritis was diagnosed in the participants using a self-report questionnaire and the following question: “Have you ever been informed by a healthcare professional that you have arthritis?” The participants were given two response options: “yes” or “no”. Participants with a “yes” response were asked to specify the type of arthritis diagnosed. This information enabled the classification of arthritis into distinct categories, including but not limited to OA, RA, and other forms of arthritis. A prior study established that the accuracy of self-reported OA and RA is deemed acceptable for large-scale studies [24].

### Covariates used in NHANES

In this study, confounding effects were considered using various covariates. The covariates examined were age (years; <60, ≥60), sex (male, female), ethnicity (Mexican American, other Hispanic, non-Hispanic White, non-Hispanic Black, other races), the poverty-to-income ratio (PIR), education level (less than high school, high school or general education diploma [GED], above high school), C-reactive protein (CRP) levels, marital status (married or living with a partner, single), body mass index (BMI), smoking habits (never, former, current), presence of hypertension (yes, no), presence of diabetes (yes, no), alcohol use (yes, no), steroid use (yes, no), statin use (yes, no), engagement in vigorous activity (yes, no), and engagement in moderate activity (yes, no). The classification of BMI consisted of three categories: normal weight (< 25 kg/m^2^), overweight (25–29.9 kg/m^2^), and obesity (≥ 30 kg/m^2^). Three categories were used to classify smoking status: never-smoker, former smoker, and current smoker. The smoking status categorization was determined by the number of cigarettes smoked by individuals in their lifetime (threshold: <100 cigarettes) and their current smoking status. Alcohol use was assessed based on whether the participant had ≥ 5 drinks every day. Steroid use and statin medication data were acquired from the medication questionnaire. The participants’ medication intake in the last 30 days was queried and categorized based on a “yes” or “no” response regarding medication use. Participants who answered “yes” were prompted to specify the medications they had used. Taking medications containing prednisone, prednisolone, methylprednisolone, or methylprednisolone acetate was categorized as steroid use [25]. Similarly, taking a statin lipid-lowering drug was categorized as statin use. For a detailed understanding of each variable employed in this study, comprehensive instructions can be found on the NHANES website.

### Statistical analysis

In this observational study, NHANES data were employed to explore the association between RC and RA using multivariate logistic regression analysis.

Mean ± standard deviation was used to report continuous variables, indicating their distribution, whereas frequencies and percentages were presented for categorical variables to offer a comprehensive overview. The RC was categorized into tertiles, and for comparative analysis, the lowest tertile (T1) served as the reference group. The comparison of RC tertile groups was performed using either the chi-squared test or Kruskal-Wallis H test for statistical analysis.

The association between RC and RA was investigated through a comprehensive multivariate logistic regression analysis. Model 1 remained unadjusted for gender, age, or ethnicity, while Model 2 incorporated adjustments for these variables. In Model 3, the Model 2 variables were considered along with marital status, education level, BMI, vigorous activity, moderate activity, hypertension, diabetes, smoking status, alcohol use, steroid use, statin use, PIR, and CRP. The age, sex, BMI, vigorous activity, moderate activity, hypertension, smoker status, and diabetes subgroups also underwent the same statistical procedures as described previously, enabling the examination of possible trends within these individuals.

The nonlinear relationship between RC and RA was comprehensively evaluated using smooth curve fitting and analysis of threshold effects. Statistical analyses were conducted utilizing R 4.1.3 and EmpowerStats 2.0, employing a predetermined significance threshold of *P* < 0.05.

## Results

**Fig. 1:**
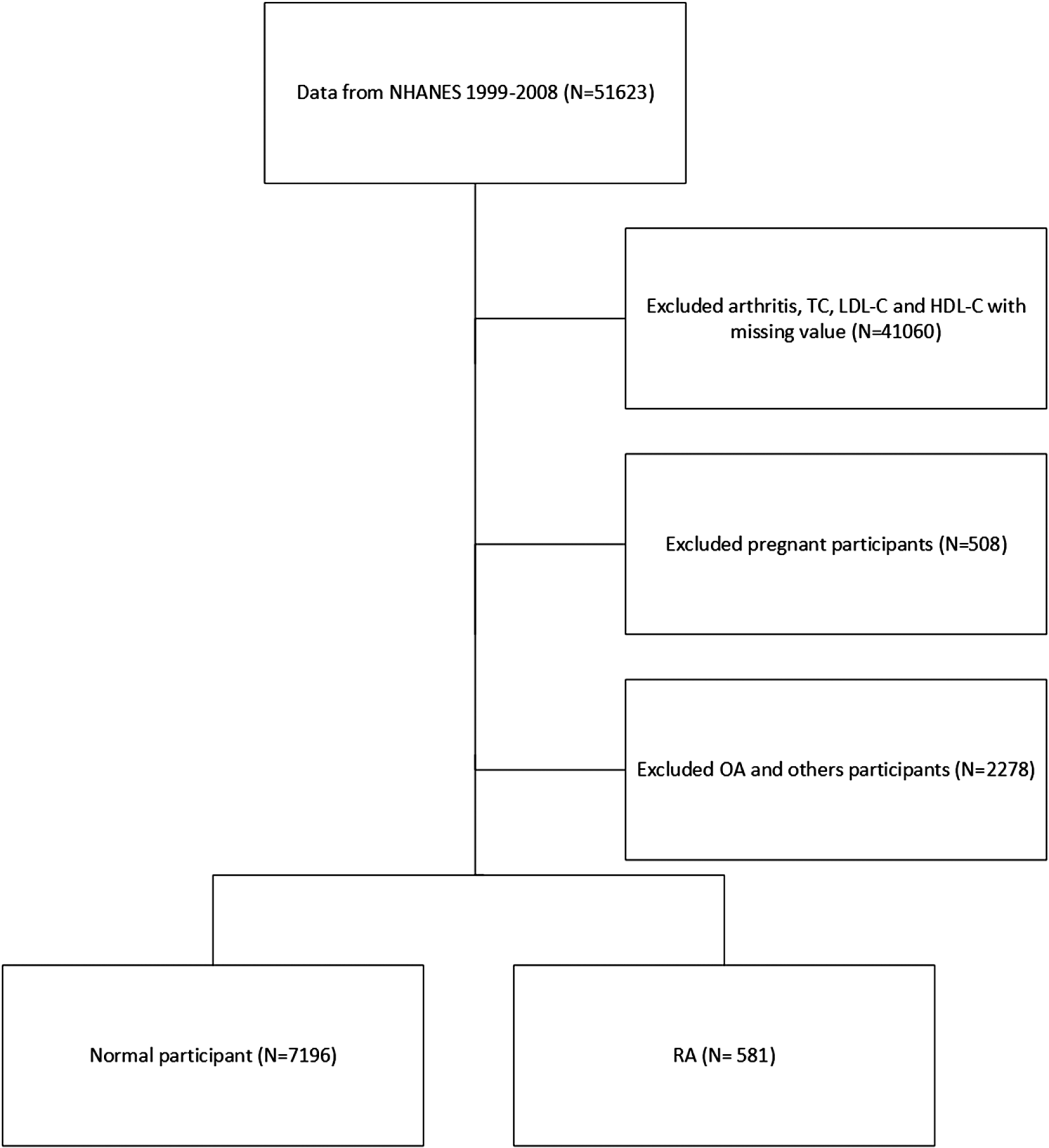
Flowchart of the participant selection from NHANES 1999–2008

After applying exclusion criteria, we excluded 41,060 participants due to insufficient arthritis and lipid data, 2278 participants due to OA or other irrelevant conditions, and 508 pregnant participants. Thus, the final study population comprised 7777 participants. Figure 1 illustrates the participant selection process.

### Participants’ baseline characteristics

The attributes of the participants with and without RA are contrasted in Table 1. The cohort included 7777 individuals who satisfied the selection criteria, where the RA prevalence was 7.47%. Among the participants, 71.9% were aged < 60 years, 52.72% were male, 62.21% were married or had a partner, 46.89% were educated above high school, 28.42% had hypertension, 52.76% were never-smokers, and 7.83% had diabetes. Regarding ethnic background, 22.53% of the participants identified as Mexican American, 6.0% were of different Hispanic origins, 46.79% were non-Hispanic Whites, 20.83% were non-Hispanic Blacks, and 3.84% belonged to other racial categories. The overall the mean values for CRP, TC, HDL-C, LDL-C, and RC were, in order, 0.44 ± 0.86 mg/dL, 5.09 ± 1.05 mmol/L, 1.37 ± 0.40 mmol/L, 3.06 ± 0.93 mmol/L, and 0.66 ± 0.35 mmol/L.

**Table 1:** Baseline characteristics of RA vs. non-RA groups

	Overall	Non-RA	RA	*P*-value
Age, years (%)				< 0.001
< 60	71.90	74.50	39.76	
≥60	28.10	25.50	60.24	
Sex (%)				< 0.001
Male	52.72	53.57	42.17	
Female	47.28	46.43	57.83	
Race (%)				< 0.001
Mexican American	22.53	22.89	18.07	
Other Hispanic	6.00	6.14	4.30	
Non-Hispanic White	46.79	46.83	46.30	
Non-Hispanic Black	20.83	20.23	28.23	
Other	3.84	3.90	3.10	
Marital status (%)				< 0.001
Married or with partner	62.21	62.90	53.75	
Single	37.79	37.10	46.25	
Education level (%)				< 0.001
Less than high school	29.68	28.71	41.65	
High school or GED	23.43	23.23	25.99	
Above high school	46.89	48.07	32.36	
BMI (%)				< 0.001
Normal weight	33.87	34.81	22.06	
Overweight	35.32	35.43	33.99	
Obese	30.81	29.76	43.95	
Vigorous activity (%)				< 0.001
Yes	30.55	31.48	18.11	
No	69.45	68.52	81.89	
Moderate activity (%)				0.001
Yes	47.30	47.81	40.66	
No	52.70	52.19	59.34	
Hypertension (%)				< 0.001
Yes	28.42	26.22	55.44	
No	71.58	73.78	44.56	
Diabetes				< 0.001
Yes	7.83	6.95	18.77	
No	92.17	93.05	81.23	
Smoker status				< 0.001
Current	23.02	22.76	26.16	
Former	24.23	23.61	31.84	
Never	52.76	53.63	42.00	
Alcohol use				0.073
Yes	17.07	16.82	20.04	
No	82.93	83.18	79.96	
Steroid use				< 0.001
Yes	1.05	0.74	4.90	
No	98.95	99.26	95.10	
Statin use				< 0.001
Yes	10.93	9.81	25.00	
No	89.07	90.19	75.00	
PIR				< 0.001
< 1	17.27	17.01	20.49	
1–3	41.82	41.20	49.62	
≥ 3	40.92	41.80	29.89	
CRP (mg/dL)	0.44 ± 0.86	0.42 ± 0.81	0.73 ± 1.31	< 0.001
TC (mmol/L)	5.09 ± 1.05	5.08 ± 1.05	5.17 ± 1.04	0.016
HDL-C (mmol/L)	1.37 ± 0.40	1.37 ± 0.40	1.38 ± 0.45	0.677
LDL-C (mmol/L)	3.06 ± 0.93	3.06 ± 0.93	3.05 ± 0.94	0.960
RC (mmol/L)	0.66 ± 0.35	0.65 ± 0.35	0.74 ± 0.38	< 0.001

The participants were categorized into tertiles according to their RC values for further analysis. Participants with RA and aged > 60 years exhibited a higher prevalence compared to those without RA. There was a greater proportion of women exhibited RA in comparison to men. A higher proportion of participants identifying as non-Hispanic Black was observed among those with RA when compared to individuals without RA. Furthermore, the participants with RA exhibited a higher prevalence of non-diabetes, obesity, non-activity, never-smoker status, and hypertension. The participants with RA had higher RC and TC values than those without RA.

Table 2 presents the participants’ baseline characteristics. Participants in T3 were more likely to be ≥60 years old, obese, and male than those in T1. Participants with higher RC had lower education, higher marital status, higher smoking rates, high blood pressure, diabetes, and higher RC. An increase in the RC level was accompanied by significantly increased RA occurrence (T1, 5.16%; T2, 7.88%; T3, 9.35%, *P* < 0.001).

**Table 2:** Participants’ baseline characteristics according to RC tertiles

	Overall	T1 (< 0.46)	T2 (0.46–0.72)	T3 (≥ 0.72)	*P*-value
Age, years (%)					< 0.001
< 60	71.90	80.86	69.13	65.81	
≥60	28.10	19.14	30.87	34.19	
Sex (%)					< 0.001
Male	52.72	45.92	54.09	58.08	
Female	47.28	54.08	45.91	41.92	
Race (%)					< 0.001
Mexican American	22.53	16.77	22.65	28.12	
Other Hispanic	6.00	5.16	5.96	6.88	
Non-Hispanic White	46.79	43.59	47.25	49.50	
Non-Hispanic Black	20.83	31.29	19.68	11.62	
Other	3.84	3.18	4.46	3.88	
Marital status (%)					< 0.001
Married or with partner	62.21	58.29	61.47	66.88	
Single	37.79	41.71	38.53	33.12	
Education level (%)					< 0.001
Less than high school	29.68	25.30	29.87	33.82	
High school or GED	23.43	21.69	23.98	24.61	
Above high school	46.89	53.01	46.15	41.56	
BMI (%)					< 0.001
Normal weight	33.87	48.86	33.27	19.59	
Overweight	35.32	29.95	36.48	39.50	
Obese	30.81	21.19	30.25	40.91	
Vigorous activity (%)					< 0.001
Yes	30.55	35.80	29.91	25.91	
No	69.45	64.20	70.09	74.09	
Moderate activity (%)					0.110
Yes	47.30	48.94	46.87	46.10	
No	52.70	51.06	53.13	53.90	
Hypertension (%)					< 0.001
Yes	28.42	19.84	29.96	35.37	
No	71.58	80.16	70.04	64.63	
Diabetes					< 0.001
Yes	7.83	4.37	7.42	11.72	
No	92.17	95.63	92.58	88.28	
Smoker status					< 0.001
Current	23.02	20.92	24.34	23.77	
Former	24.23	19.68	24.03	28.92	
Never	52.76	59.39	51.63	47.31	
Alcohol use					< 0.001
Yes	17.07	277 (13.48%)	373 (17.65%)	423 (19.96%)	
No	82.93	1778 (86.52%)	1740 (82.35%)	1696 (80.04%)	
Steroid use					0.567
Yes	1.05	23 (0.90%)	31 (1.20%)	27 (1.04%)	
No	98.95	2543 (99.10%)	2556 (98.80%)	2559 (98.96%)	
Statin use					< 0.001
Yes	10.93	172 (6.70%)	288 (11.13%)	386 (14.93%)	
No	89.07	2394 (93.30%)	2299 (88.87%)	2200 (85.07%)	
PIR					0.229
< 1	17.27	412 (17.38%)	409 (16.87%)	423 (17.55%)	
1–3	41.82	950 (40.07%)	1030 (42.49%)	1033 (42.86%)	
≥ 3	40.92	1009 (42.56%)	985 (40.64%)	954 (39.59%)	
CRP (mg/dL)	0.44 ± 0.86	0.34 ± 0.71	0.48 ± 0.98	0.50 ± 0.87	< 0.001
TC (mmol/L)	5.09 ± 1.05	4.66 ± 0.93	5.12 ± 0.97	5.47 ± 1.08	< 0.001
HDL-C (mmol/L)	1.37 ± 0.40	1.56 ± 0.42	1.37 ± 0.37	1.18 ± 0.31	< 0.001
LDL-C (mmol/L)	3.06 ± 0.93	2.76 ± 0.81	3.17 ± 0.89	3.23 ± 1.01	< 0.001
RC (mmol/L)	0.66 ± 0.35	0.34 ± 0.08	0.58 ± 0.08	1.06 ± 0.30	< 0.001
RA (%)					< 0.001
Yes	7.47	5.16	7.88	9.35	
No	92.53	94.84	92.12	90.65	

### Associations between RC and RA

**Fig. 2:**
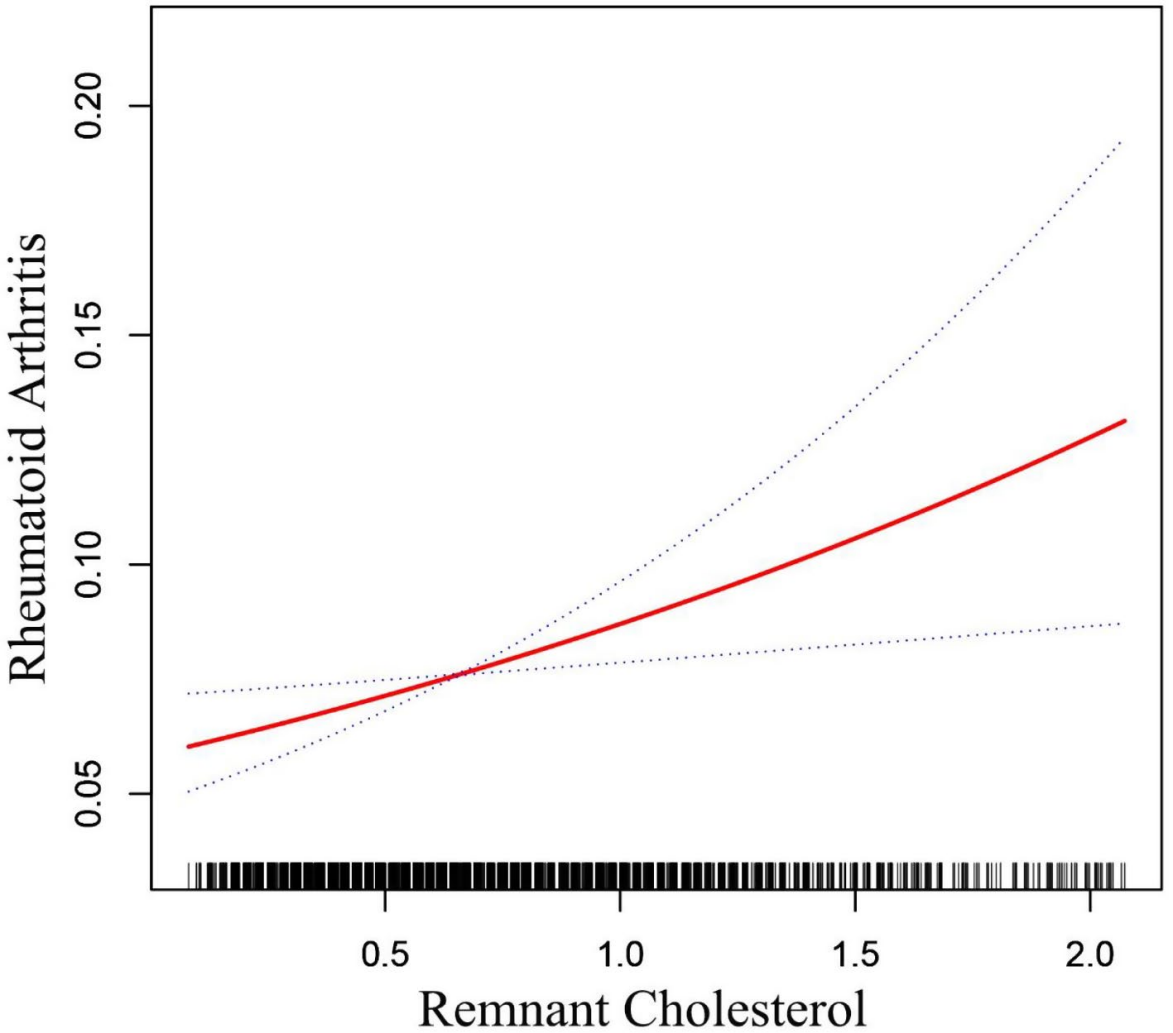
Relationship between RC and RA

The smooth curve fitting confirmed the linear positive relationship between RC and RA (Fig. 2). Table 3 presents a comprehensive overview of the multivariate logistic regression analysis results. Both the crude (OR = 1.91; 95% CI: 1.54–2.37; *P* < 0.0001) and adjusted models (OR = 1.91; 95% CI: 1.53–2.45; *P* < 0.0001) consistently demonstrated a strong positive correlation between RC and RA. The relationship between RC and RA remained significant and positive even after all variables had been adjusted in Model 3 (OR = 1.54; 95% CI: 1.11–2.13; *P* = 0.0092). According to this finding, the probabilities of RA increased by 54% for every unit rise in RC. Model 3 demonstrated that RA occurrence was increased in T3 compared to T1 (OR = 1.47; 95% CI: 1.07–2.01; *P* < 0.016). Furthermore, TC, HDL-C, LDL-C, and RA were exhibit a statistically significant correlation.

**Table 3:** The OR and 95% CI for RA according to RC tertiles

	OR (95% CI), *P*-value		
	Model 1^1^	Model 2^2^	Model 3^3^
	*n* = 7,777	*n* = 7,777	*n* = 4,500
RA			
RC	1.91 (1.54, 2.37), < 0.0001	1.94 (1.53, 2.45), < 0.0001	1.54 (1.11, 2.13), 0.0092
Categories			
T1	Reference	Reference	Reference
T2	1.57 (1.25, 1.97), < 0.0001	1.46 (1.16, 1.85), 0.0015	1.13 (0.82, 1.55), 0.4599
T3	1.89 (1.52, 2.36), < 0.0001	1.83 (1.45, 2.31), < 0.0001	1.47 (1.07, 2.01), 0.0160
*P* for trend	< 0.0001	< 0.0001	0.0097
TC	1.09 (1.01, 1.18), 0.0330	0.99 (0.91, 1.07), 0.7322	1.04 (0.92, 1.16), 0.5361
LDL-C	0.99 (0.91, 1.09), 0.8761	0.95 (0.87, 1.05), 0.3201	1.00 (0.87, 1.14), 0.9666
HDL-C	1.08 (0.88, 1.32), 0.4837	0.69 (0.55, 0.87), 0.0016	0.91 (0.66, 1.24), 0.5441

In sensitivity analysis, RC was converted from a continuous variable to a categorical variable (tertiles)

OR: Odds ratio; 95% Cl: 95% confidence interval

^1^Model 1: No covariates were adjusted

^2^Model 2: Adjusted for sex, age, and race

^3^Model 3: Adjusted for sex, age, race, marital status, education level, BMI, vigorous activity, moderate activity, hypertension, diabetes, smoker status, alcohol use, steroid use, statin use, PIR, and CRP

### Subgroup analysis and interaction testing

**Fig. 3:**
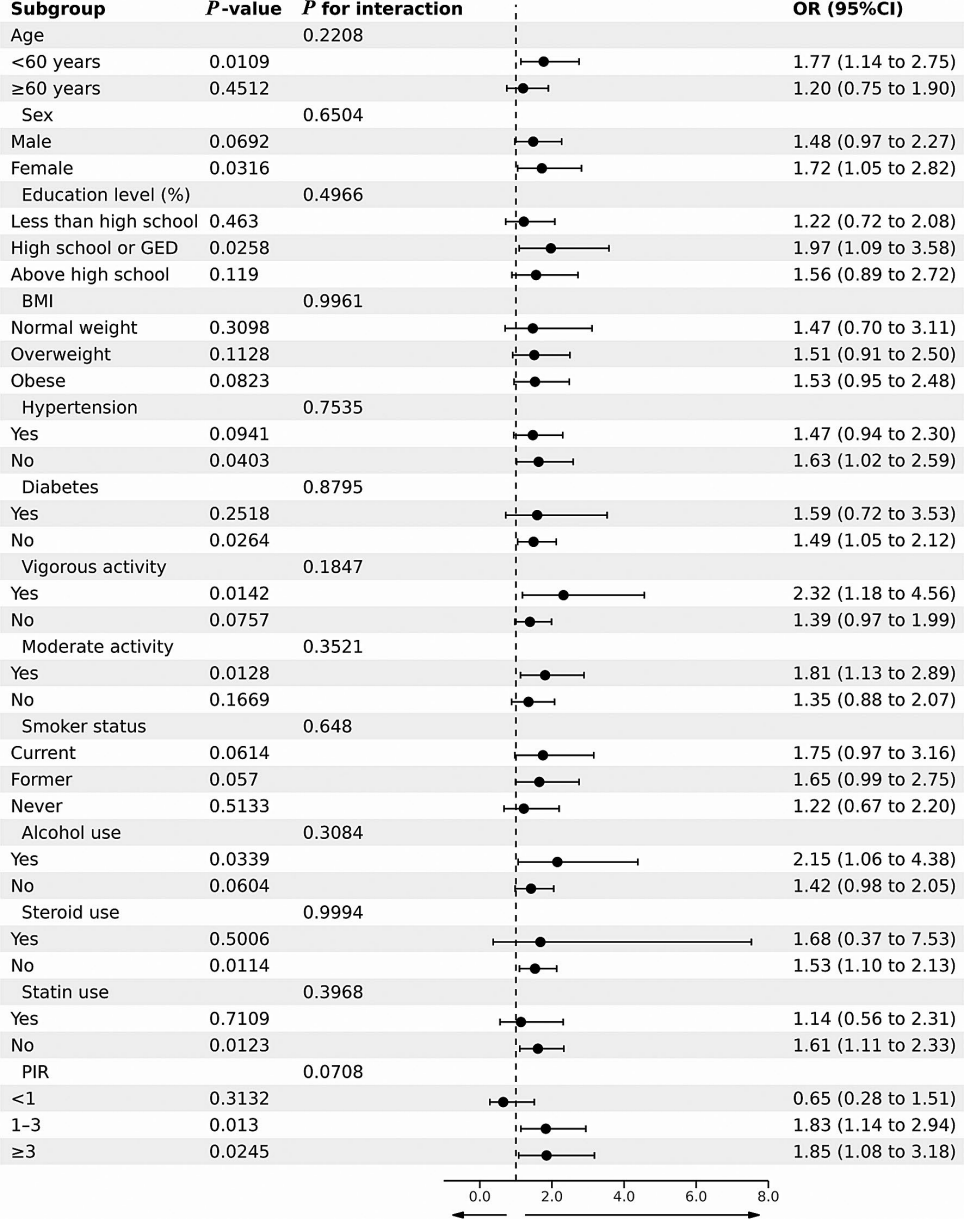
The OR of RA subgroups based on an increased RC index

Figure 3 displays the outcomes of a stratified analysis performed in the subgroup investigation, taking into account variables such as sex, age, education level, BMI, vigorous activity, moderate activity, hypertension, diabetes, and smoker status. The interaction test demonstrated that those subgroups had no significant effect on this connection (all *P* for interaction > 0.05). When the age was <60 years, each additional unit of RC raised the chance of developing RA by 77% (OR = 1.77, 95% CI: 1.14–2.75, *P* = 0.0109). RC and RA were correlated in females (OR = 1.72, 95% CI: 1.05–2.82, *P* = 0.0316) but not in males. Similarly, the lack of diabetes enhanced the influence of RC on the probability of RA (OR = 1.49, 95% CI: 1.05–2.12, *P* = 0.0264), exceeding the effect observed in individuals with diabetes. The association between RC and RA was evident in individuals who engaged in vigorous (OR = 2.32, 95% CI: 1.18–4.56, *P* = 0.0142) and moderate activity (OR = 1.81, 95% CI: 1.13–2.89, *P* = 0.0128), in contrast to those without these physical activity levels. The absence of steroid use enhanced the influence of RC on the probability of RA (OR = 1.53, 95% CI: 1.10–2.13, *P* = 0.0114), exceeding the effect observed in individuals who used steroids (OR = 1.53, 95% CI: 1.10–2.13, *P* = 0.5006). The absence of statin use amplified the effect of RC on the likelihood of RA (OR = 1.61, 95% CI: 1.11–2.33, *P* = 0.0123), surpassing the influence observed in individuals with statin use.

## Discussion

This cross-sectional study involving 7777 eligible participants identified a positive association between RC and RA. This correlation persisted even after rigorous triangulation of RC and meticulous adjustment for pertinent confounders in the fully adjusted model. There was no correlation between TC, LDL-C, and HDL-C and RA according to the logistic regression research. The findings emphasized the crucial role of RC in comprehending the odds of having RA.

Extensive research utilizing the NHANES database has progressively identified RC as a biomarker of various diseases. Zhang et al. [26] found some sort of correlation between log-transformed RC levels and cardiovascular mortality among 19,650 adults (Hazard Ratio = 2.82; 95% CI: 1.17–6.81). Shi et al. [19] examined 4963 adults and established an autonomous link between RC and hypertension regardless of LDL-C levels. This correlation persisted even when considering elevated TG levels, hinting at the potential implication of other lipoproteins beyond ApoB. Xie et al. [27] examined 1377 patients aged ≥ 60 years and determined that for every 1 mmol/L increase in RC, the probability of a patient achieving a higher Alzheimer’s Disease total score decreased by approximately 26%. This result suggested that lower RC levels are linked to enhanced verbal learning and memory functions, implying that reducing RC levels could mitigate cognitive impairment in older adults. Wang et al. [28] surveyed 8263 adults and observed a positive association between RC concentration and depression, particularly among participants aged < 60 years, men, those with BMI < 30 kg/m^2^, and individuals with diabetes. Chen et al. [29] investigated 3370 participants and identified a nonlinear positive relationship between RC and non-alcoholic fatty liver disease, with an inflection point at 0.96 mmol/L. Hu et al. [30] scrutinized 11,838 participants and detected a notable correlation between elevated serum RC levels and the degree of frailty in middle-aged and older adults, suggesting a nonlinear dose–response relationship (nonlinear *P* = 0.011). This accumulating body of evidence demonstrates that RC is not solely applicable to patients with CVD but may also be associated with various other diseases. Nevertheless, the possible connection between RC and RA has not been examined yet.

This study constitutes a population-based investigation aimed at elucidating the relationship between RA and RC. Prior research indicated that individuals with RA often exhibit distinct lipid profiles compared to those without RA. Studies examining lipid levels in RA patients yielded conflicting conclusions. Despite the observed increased cardiovascular disease risk in RA, the levels of TC, LDL-C, and HDL-C typically decrease, especially during states of heightened inflammation. This phenomenon is commonly referred to as the lipid paradox. Kim et al. [31] examined 46 Korean participants and reported that RA patients had normal TC and LDL-C levels. However, the RA group demonstrated a notable reduction in HDL-C levels and the ratio of HDL-C/TC and substantially elevated blood TG levels. Given the limited sample size, these findings should be interpreted cautiously, considering the limitations in generalizability. Curtis et al. [32] conducted a comprehensive study involving 30,586 RA patients and 107,534 OA patients in the United States, revealing significant disparities in lipid profiles and associated health conditions between the two cohorts. The RA patients exhibited lower mean TC, LDL-C, and TG levels, with slightly higher HDL-C levels than the OA patients. Furthermore, According to the ATP-III lipid classification standards, fewer RA patients exhibit borderline high or high levels of TC and LDL-C. While exploring differences in blood lipid metabolism in RA patients using OA as a control group is of certain significance, studying these differences in the general population is even more meaningful. A Mendelian randomization study produced findings similar to those of the large population-based study. Kasher et al. [33] performed a Mendelian randomization analysis revealed complex and contradictory relationships between lipid factors and RA. Importantly, their analysis did not suggest a causal link between RA and alterations in lipid factors. Zhang et al. [34] used Genome wide association study data and demonstrated complex and sometimes contradictory relationships between lipid factors and RA. However, these analyses did not establish a causal link between RA and lipid factor alterations. Their results did not provide evidence of a causal effect of RA on TC, LDL-C, or HDL-C levels. While the abovementioned studies used various research methods on different populations and yielded somewhat contradictory results, they underscore the importance of blood lipid markers in assessing RA.

The precise mechanism underpinning the link between RC and RA remains elusive. The relationship between lipid metabolism and RA is complex, possibly arising from both inflammatory mechanisms and altered cholesterol metabolism. The following mechanisms and factors might explain the positive correlation between RC levels and RA. Hypercholesterolemia directly triggers cholesterol accumulation in immune cells such as macrophages, which increase the inflammation level by amplifying Toll-like receptor inflammatory signals and activating the inflammasome, and promote the production of other immune cells, such as neutrophils and monocytes [35, 36]. In turn, excessive inflammatory signal activation caused by high cholesterol levels reduces cellular cholesterol efflux, aggravating cholesterol accumulation and creating a vicious cycle [35]. This abnormal immune environment greatly increases the RA risk. Additionally, the main cytokines that drive the inflammatory process of RA, including tumor necrosis factor α and interleukin-6, are crucial in modifying lipid metabolism. These cytokines induce changes in the subcomponents and structure of HDL-C particles, compromising their anti-atherosclerosis function. This alteration promotes LDL-C oxidation and contributes to the formation of atherosclerotic plaques.

The malfunctioning of HDL can worsen abnormalities in LDL metabolism, increasing the susceptibility to CVD. This heightened risk of CVD is a prominent factor contributing to the leading cause of death among individuals with RA. Genetic factors are important in RA development, and some genes are closely related to cholesterol metabolism. The susceptibility genes associated with RA) including *TRAF1/C5*, *STAT4*, and *HLA-DRB1-SE*, not only contribute to RA but also impact lipid metabolism in RA patients, thereby elevating the risk of CVD [37]. Furthermore, mice with RA had defective cholesterol metabolism due to the downregulated expression of the cholesterol effector genes *Apoe*, *Abca1*, and *Abcg1* [38]. This cholesterol homeostasis disturbance indicates increased risk of CVD in RA patients. Furthermore, genetic susceptibility to dysregulation of LDL-C is linked to an elevated risk of RA. This association may explain why individuals with RA are more susceptible to cardiovascular incidents [39]. Unhealthy diet and lifestyle habits, such as high-fat diets, physical inactivity, and obesity, are strongly associated with high cholesterol levels, which are also RA risk factors [40–42]. A healthy diet and lifestyle confer a wide range of benefits, such as lower cholesterol levels and reduced risk of many diseases, including RA and CVD. Some cholesterol-reducing drugs might be associated with a reduced RA risk. Statins are widely used for treating hypercholesterolemia and were recently proven to regulate immune cells by inhibiting T cell proliferation, activation, and differentiation [43, 44], providing a theoretical basis for statin treatment of autoimmune diseases at the cellular level. TG and RC levels tend to decrease concurrently with statin therapy aimed at reducing LDL-C levels [45, 46]. Compared with the placebo group, statin-treated patients achieved significant remission of RA, indicated by the improved disease activity score and inflammatory indicators [47], suggesting the multi-functionality of statins in RA treatment and their broad prospects. Furthermore, RA drugs significantly influence lipid levels. Glucocorticoids are drugs commonly used to impede RA progression through their powerful anti-inflammatory effects. Nevertheless, glucocorticoids exert an influence on CVD risk in RA patients, manifesting in bidirectional effects on lipids. Short-term, low-dose glucocorticoid treatment may contribute to the enhancement of HDL-C levels [48], but sustained glucocorticoid use leads to fat accumulation in the liver, triggering an increase in the RC components TG and VLDL [49]. Disease-modifying anti-rheumatic drugs exerted a similarly pronounced effect on cholesterol metabolism in RA. Methotrexate promotes the transfer of intracellular cholesterol to the extracellular compartment to produce an anti-atherosclerotic effect [50]. Contrastingly, some studies disagreed, suggesting that methotrexate increases TC and TG levels [51].

Ultimately, the correlation between RC levels and RA likely stems from combined elements that include the immune response, inflammation, genetics, lifestyle habits, and medication effects. Therefore, a more profound comprehension of this correlation can be acquired using additional clinical and molecular biology investigations to ascertain the precise mechanism.

### Strengths and limitations

The extra proof this study provided regarding the beneficial link between RC and RA enhanced the body of literature. The utilization of the NHANES database facilitated the examination of a substantial and representative cohort, thereby improving the generalizability of the study outcomes. Moreover, this study augmented the comprehension of RC as a viable marker for pinpointing individuals predisposed to RA, highlighting the significance of lipid metabolic disorder in RC pathogenesis.

Nevertheless, it is essential to acknowledge the limitations inherent in this study. First, the study design was cross-sectional and had limited ability to establish causal relationships between RC and RA. Longitudinal or prospective studies would be essential to elucidate the temporal aspects of this association. Second, the RA diagnosis was obtained through NHANES self-report questionnaire data and lacked a more detailed classification of arthritis types. Third, data on medications that could influence RA and RC were not fully included. Furthermore, unmeasured confounding variables or residual confounding might have influenced the observed associations despite adjustment for numerous covariates. Finally, limitations within the database hindered the inclusion of data on all factors influencing RA.

## Conclusion

This study revealed a distinct positive correlation between RC and RA within a diverse US demographic. The findings significantly contribute to the growing evidence supporting the clinical utility of RC as a predictive index for RA, offering valuable insights for the development of early intervention strategies in populations susceptible to RA. The future aim is to explore advanced statistical techniques or alternative study designs to enhance control over confounding variables. Furthermore, the authors plan to broaden the scope of variables influencing RA by fostering collaborations and using supplementary datasets.

## Data Availability

Data are available upon request to the corresponding author.

## Abbreviations

BMI: Body mass index
CVD: Cardiovascular disease
CI: Confidence interval
CRP: C-reactive protein
GED: General education diploma
HDL-C: High-density lipoprotein cholesterol
LDL-C: Low-density lipoprotein cholesterol
NHANES: National Health and Nutrition Examination Survey
OA: Osteoarthritis
OR: Odds ratio
PIR: Poverty-to-income ratio
RA: Rheumatoid arthritis
RC: Remnant cholesterol
TC: Total cholesterol
TG: Triglycerides
VLDL: Very low-density lipoprotein
